# In vitro comparison of the adsorption of inflammatory mediators by blood purification devices

**DOI:** 10.1186/s40635-018-0177-2

**Published:** 2018-05-04

**Authors:** Benjamin Malard, Corine Lambert, John A. Kellum

**Affiliations:** 1 R&D Department, Gambro Industries, 7 avenue Lionel Terray, 69330 Meyzieu, France; 2 Center for Critical Care Nephrology, Department of Critical Care Medicine, University of Pittsburgh, 604 Scaife Hall, 3550 Terrace Street, Pittsburgh, PA 15261 USA

**Keywords:** Adsorption, Blood purification, Cytokines, Endotoxins, oXiris, Septic shock, Removal rate

## Abstract

**Background:**

Septic shock, a leading cause of acute kidney injury, induces release of pro-/anti-inflammatory mediators, leading to increased mortality and poor renal recovery. This is the first in vitro study directly comparing three single-use blood purification devices in terms of removing sepsis-associated mediators and endotoxins.

**Methods:**

In vitro hemoperfusion was performed using oXiris^®^, CytoSorb^®^, and Toraymyxin^®^. Heparinized human plasma from healthy volunteers was pre-incubated with pathologic quantities of inflammatory mediators and filtered in a closed-loop circulation model for 2 h. For each device, the removal of 27 inflammatory mediators was measured over time. Endotoxin removal mediated by oXiris and Toraymyxin was assessed using hemoperfusion over 6 h.

**Results:**

Endotoxin (lipopolysaccharide) removal was most rapid with Toraymyxin; mean adsorptive clearance over the first 30 min was ~ 20 ml/min vs ~ 8 ml/min with oXiris (*p* < 0.05). There was minimal endotoxin removal with CytoSorb (1 ml/min). At 120 min, there was no significant difference between the endotoxin removal rates using oXiris (mean ± standard deviation, 68.0 ± 4.4%) and Toraymyxin (83.4 ± 3.8%); both were significantly higher vs CytoSorb (− 6.3 ± 4.9%; *p* < 0.05). Total removal with oXiris was 6.9 μg vs 9.7 μg for Toraymyxin, where the total lipopolysaccharide quantity introduced was approximately 15.8 μg. Removal rates of pro-/anti-inflammatory cytokines and other inflammatory mediators were similar between oXiris and CytoSorb and were higher with CytoSorb and oXiris vs Toraymyxin. Granulocyte colony-stimulating factor was only effectively adsorbed by CytoSorb (99.4%). Differences were detected between the adsorption mechanism of the devices; binding to oXiris was mainly ionic, while CytoSorb was hydrophobic. No specific protein adsorption was found qualitatively with Toraymyxin.

**Conclusions:**

Adsorption rate kinetics varied for individual inflammatory mediators using the three blood purification devices. Mechanisms of adsorption differed between the devices. oXiris was the only device tested that showed both endotoxin and cytokine removal. oXiris showed similar endotoxin adsorption to Toraymyxin and similar adsorption to CytoSorb for the removal of other inflammatory mediators. Differences in device removal capacities could enable treatment to be more tailored to patients.

**Electronic supplementary material:**

The online version of this article (10.1186/s40635-018-0177-2) contains supplementary material, which is available to authorized users.

## Background

**Fig. 1:**
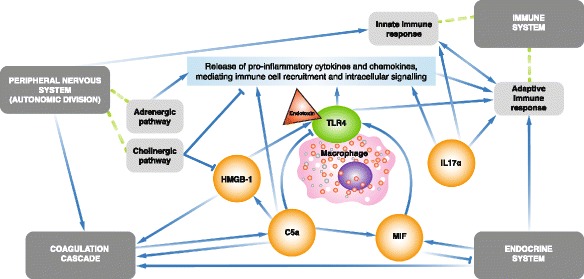
Centers of activity in the inflammatory network driving sepsis. *Abbreviations*: *C5a* complement 5a, *HMGB-1* high-mobility group box 1 protein, *IL-17A* interleukin-17A, *MIF* macrophage migration inhibitory factor, *TLR4* Toll-like receptor 4 [6]

Patients with acute kidney injury (AKI) have raised serum levels of inflammatory mediators, regardless of the cause [1], and are associated with poor outcomes [2]. Septic shock is a leading cause of AKI [2], accounting for half or more of the cases in intensive care units [3]. The pathophysiology of sepsis and septic shock is not completely understood but is known to involve the release of both pro- and anti-inflammatory mediators [4]. Endotoxins are important in the pathogenesis of septic shock, whereby infection triggers a systemic inflammatory response, resulting in release of pro- and anti-inflammatory cytokines [4–6] (Fig. 1). Pro-inflammatory immune responses are thought to be responsible for the tissue damage that occurs in severe sepsis, while anti-inflammatory responses are implicated in the enhanced susceptibility to secondary infections [7]. Sepsis can also involve the activation of coagulation pathways [4], leading to a higher risk of death [8].

Due to the complexity of the immune response, it is unlikely that neutralization of a single indicator would be effective in modulating the inflammatory response, and it is important to look more widely. Several blood purification devices are available that can remove both exogenous and endogenous inflammatory mediators [5]. Such tools include oXiris^®^, Toraymyxin^®^, and CytoSorb^®^. oXiris is a hollow fiber acrylonitrile and methalylsulfonate (AN69) membrane that removes larger molecular weight molecules by membrane binding [5, 9]. Approved first in Europe in 2009, its initial CE-marked indication was extended in 2017 for patients who require blood purification, including those requiring continuous renal replacement therapy (CRRT), and in conditions with excessive endotoxin and inflammatory mediator levels [5, 9]. Approved first in Japan in 1994 and qualifying for CE marking in 1998, Toraymyxin is indicated for use in the treatment of patients with sepsis or septic shock caused by gram-negative bacteria by selectively removing endotoxins from the circulating blood of patients [10]. It consists of a cartridge containing polystyrene-derived woven fibers with the antibiotic polymyxin B immobilized on the surface [5, 10]. Approved first in Europe in 2011 with an indication for use for patients with conditions where excessive cytokine levels exist, CytoSorb is a CE-marked device containing polymer beads to adsorb cytokines that is used in blood pump circuits [5, 11].

Devices such as oXiris can be used on a continuous basis for up to 72 h, although it is recommended that the set is changed every 24 h [9]. Therefore, the ability to retain endotoxins over long periods without the device becoming saturated is of interest. It is possible that differences in the design of these devices may mean that they remove mediators differently (in terms of quantity removed and kinetics). Thus, identifying differences between them could be an important step towards more tailored therapies.

The studies described above have investigated the adsorptive capabilities of these devices for inflammatory mediators such as endotoxins and cytokines. However, we report the first study to directly compare multiple devices across a large spectrum of inflammatory mediators. In this in vitro study, we compared the three single-use blood purification devices in terms of removal of a large panel of sepsis-associated mediators between oXiris, Toraymyxin, and CytoSorb. In addition, we also assessed the removal of endotoxins over a 6-h period by oXiris and Toraymyxin.

## Methods

### Part 1: Short-term experimental set-up

A total of *n* = 3 in vitro experimental hemoperfusions were performed comparing oXiris (Baxter, Meyzieu, France), CytoSorb (CytoSorbents Corporation, New Jersey, USA), Toraymyxin (Toray Industries, Tokyo, Japan; Table 1), and a control (tubing circuit without the hemofilter). Devices were primed in accordance with their instructions for use. For each circulation, a total volume of 2-l heparinized fresh frozen human plasma (EFS, Strasbourg, France) was pooled from healthy volunteers and pre-incubated with pathological quantities of inflammatory mediators (Additional file 1: Table S1). The pool was then divided into four reservoirs of 500 ml each and filtered in a closed-loop circulation model for 2 h (Additional file 2: Table S2).

**Table 1:** Characteristics of investigated blood purification devices

Device	Membrane material	Structure	Sterilization mode
oXiris (Baxter)	Copolymer of AN69-coated with PEI and unfractionated heparin	Hollow fiber, symmetric	EtO
Toraymyxin (Toray Industries)	PS-based composite woven fiber with immobilized polymyxin B	Woven fibers	Steam
CytoSorb (CytoSorbents)	PSDVB copolymer beads, which are highly porous and covered with PVP	Microporous beads	Gamma Irradiation

*Abbreviations*: *AN69* acrylonitrile and methalylsulfonate, *EtO* ethylene oxide, *PEI* polyethylenimine, *PSDVB* polystyrene divinylbenzene, *PS* polystyrene, *PVP* polyvinylpyrrolidone

### Measurements (short-term sampling)

Respective concentrations of 27 different inflammatory mediators were measured over time to study the removal capabilities of each device (Additional file 3: Figure S1). Plasma pool samples were taken at times (t) t0, t5, t10, t30, t60, and t120 minutes.

### Laboratory analysis

Qualitative evaluation of the proteins adsorbed onto each device was performed by four successive elutions of the membrane with different buffers and subsequent electrophoretic analysis of the elutes. Each device was rinsed with 2 l saline and eluted successively with four different buffers, either 500 ml of an ionic (NaCl 1 M or glycine buffer [HCl] 100 mM [pH 2.25]) or a hydrophobic (glycine buffer [NaOH] 50 mM [pH 11] or SDS 2% in water) desorption mechanism. Devices were rinsed with 1 l of water between the use of each novel desorption media. Samples (> 25 ml) were collected from each desorption circulation and lyophilized at − 80 °C before electrophoretic patterns migration. Samples were diluted (1:2) with a sample buffer (100 M Tris-HCl, pH 6.8, 1% SDS, 4% 2-mercaptoethanol, 0.02% brilliant blue G, and 24% glycerol) (S3047, Sigma) and heated at 100 °C for 4 min. Electrophoretic protein migration was performed using both Tris-tricine (1060–26,000 Da) and Tris-glycine (12,300–78,000 Da) gels, and protein bands were revealed by silver nitrate staining.

Endotoxin (lipopolysaccharide [LPS]) concentrations were measured using the limulus amoebocyte lysate chromogenic method (K-QCL-Lonza assay; LONZA Group Ltd., Basel, Switzerland), concentrations of complement factors C3a and C5a were measured using Singleplex with enzyme-linked immunosorbent assay from commercial kits (Quidel Corporation, San Diego, USA), and high-mobility group box 1 protein (HMGB-1; IBL International GMBH, Hamburg, Germany), and all other markers were measured with MILLIPLEX^®^ Multiplex Assays (Merck Millipore, St-Quentin-en-Yvelines, France) using Luminex^®^ Magpix.

### Part 2: Long-term experimental set up

In a separate experiment using the same closed-circuit loop, a hemoperfusion procedure was performed on oXiris, Toraymyxin, and a control tubing over 6 h to document the removal of endotoxins over time. To investigate the saturation of the membrane, three circulations of heparinized human plasma media incubated with LPS were performed successively, using the same device (2 h each), and the LPS concentration was measured in duplicate using the K-QCL-Lonza assay.

### Data analysis

Marker removal during the hemofiltration procedure is due exclusively to an adsorption mechanism. The following equation was used to calculate the removal ratio of each marker at the end of the session (i.e., RR_Ad_, %_s_): RR_Ads_ = {*C*_B(0)_ − *C*_B(120)_}/*C*_B(0)_, where *C*_B(0)_ is the concentration in the plasma reservoir at baseline (t0), and *C*_B(120)_ is the concentration at the end of the session (t120).

The mean adsorption clearance for each mediator was simulated using a model of monoexponential decay (limited to the first 30 min of circulation), employing the two equations: *C*_B(30)_ = *C*_B(0)_ e^–*k t*^ and CL_(30)_ = *k ×* V, where *C*_B(0)_ and *C*_B(30)_ were respective concentrations in the plasma reservoir at t0 and t30, respectively. Here, *k* is the slope of decay, *t* is the time, *V* is the plasma reservoir volume, and CL_(30)_ is the mean adsorptive clearance over 30 min of circulation.

The adsorption isotherm was represented using the equation: Quantity_adsorbed_ = RR_Ads_ × Quantity_introduced_.

Data analyses performed included the removal capabilities of each investigated blood purification device overall and for each tested molecule, as well as kinetic removal profiles of endotoxins, pro-inflammatory cytokines, anti-inflammatory cytokines, and other inflammatory mediators over 120 min. Concentrations of LPS measured at the start of hemofiltration procedure and over time, and the quantity of LPS adsorbed, were calculated following long-term sampling using the above methods.

### Statistical analysis

Differences were analyzed using Kruskal-Wallis non-parametric testing using Minitab software (Minitab Inc., State College, USA). The *Z* value determined how the mean for each group differed from the mean of all observations, with *p* values < 0.05 indicating a significant difference.

## Results

### Endotoxin removal

**Fig. 2:**
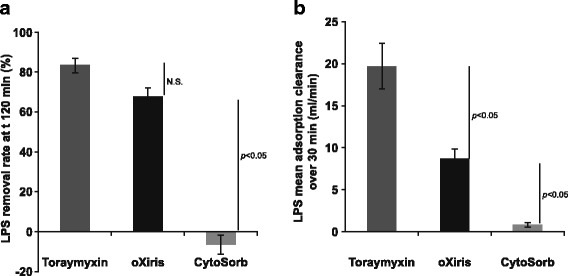
LPS removal with oXiris, Toraymyxin, and CytoSorb. **a** LPS removal rate at t120 min. **b** LPS mean adsorption clearance over 30 min. *Abbreviations*: *LPS* lipopolysaccharide, *N.S*. not significant

Adsorption of endotoxin was observed with oXiris and Toraymyxin but not with CytoSorb (Fig. 2). Endotoxin removal was most rapid with Toraymyxin (Additional file 3: Figure S1); mean absorptive clearance over the first 30 min was ~ 20 ml/min vs ~ 8 ml/min with oXiris (*p* < 0.05). Over the first 30 min, the mean adsorption clearance was greatest with CytoSorb, followed by oXiris (Fig. 2). Removal of LPS was not observed with CytoSorb at 1 ml/min. At t120, there was no significant difference between the LPS removal rates (RRs) using oXiris (mean ± standard deviation, 68.0 ± 4.3%) and Toraymyxin (83.4 ± 3.8%); rates with both were significantly higher vs CytoSorb (− 6.3 ± 4.9%; *p* < 0.05).

**Fig. 3:**
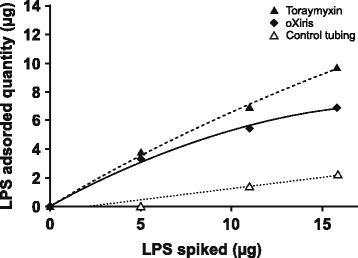
LPS adsorption isotherm obtained with oXiris and Toraymyxin blood purification devices. *Abbreviation*: *LPS* lipopolysaccharide

High endotoxin removal capacities were confirmed with oXiris and Toraymyxin over 6 h. The total removal quantity over 6 h with oXiris was 6.9 vs 9.7 μg for Toraymyxin, where the total LPS quantity introduced was approximately 15.8 μg (Fig. 3). For both oXiris and Toraymyxin, adsorption of LPS followed the Langmuir isotherm model [12]. Faster saturation of the LPS-adsorptive capacity was observed for oXiris than for Toraymyxin (Fig. 3). For oXiris and Toraymyxin, respectively, the calculated RR (%) after circulation 1 was 66.6 vs 75.9%, after circulation 2 was 35.0 vs 51.6%, and after circulation 3 was 30.5 vs 58.2%.

### Adsorption of different inflammatory mediators

**Fig. 4:**
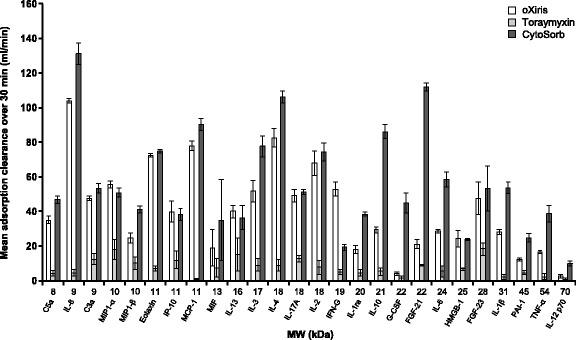
Mean adsorption clearances over 30 min for the inflammatory mediators included in the study. *Abbreviations*: *C3a* complement 3a, *C5a* complement 5a, *FGF* fibroblast growth factor, *G-CSF* granulocyte-colony stimulating factor, *HMGB-1* high-mobility group box 1 protein, *IFN* interferon, *IL* interleukin, *IP* interferon-induced protein, *MCP* monocyte chemoattractant protein, *MIF* macrophage migration inhibitory factor, *MIP* macrophage inflammatory protein, *PAI* plasminogen activator inhibitor, *TNF* tumor necrosis factor, *Ra* receptor agonist, *α* alpha, *β* beta, *γ* gamma

Adsorption of inflammatory mediators is represented for each device as the mean calculated removal rates at t120 min (Table 2). The mean adsorptive clearances after 30 min of circulation are represented as a function of tested molecules ranked according to their respective molecular weight (Fig. 4).

**Table 2:** Removal capabilities of investigated blood purification device per mediator. Means expressed ± SD

	Mediators	Removal rates (RR%) at 120 min			
		Control tubing	oXiris	CytoSorb	Toraymyxin
Pro-inflammatory cytokines	IL-3	3.9 (± 1.4)	99.3 (± 0.0)	99.4 (± 0.0)	70.2 (± 11.1)
	IP-10	16.6 (± 17.4)	99.3 (± 0.3)	99.1 (± 0.2)	68.6 (± 11.9)
	IL-17α	16.7 (± 7.8)	98.7 (± 0.4)	97.6 (± 0.3)	74.0 (± 6.2)
	MIP-1α	2.9 (± 3.1)	97.3 (± 0.4)	97.3 (± 0.4)	91.0 (± 0.4)
	MIP-1β	4.7 (± 2.9)	91.5 (± 1.2)	92.4 (± 0.0)	70.3 (± 9.0)
	HMGB-1	8.4 (± 3.2)	89.5 (± 0.4)	91.8 (± 0.9)	61.5 (± 1.9)
	IL-8	4.6 (± 8.0)	100 (± 0.0)	100 (± 0.0)	34.5 (± 13.1)
	IFN-γ	7.9 (± 8.8)	99.5 (± 0.3)	95.7 (± 0.6)	37.4 (± 8.3)
	Eotaxin	14.3 (± 9.1)	99.1 (± 0.1)	99.0 (± 0.0)	42.2 (± 7.9)
	IL-6	5.2 (± 9.3)	93.5 (± 1.4)	99.6 (± 0.1)	41.8 (± 14.6)
	MIF	14.3 (± 5.9)	78.0 (± 24.4)	83.0 (± 20.2)	45.1 (± 13.8)
	MCP-1	6.0 (± 4.1)	100 (± 0.0)	100 (± 0.0)	11.3 (± 4.4)
	TNF-α	11.8 (± 12.5)	90.1 (± 2.2)	98.4 (± 0.2)	17.9 (± 9.2)
	IL-1β	8.2 (± 4.5)	86.8 (± 1.0)	97.2 (± 0.0)	15.0 (± 13.3)
Anti-inflammatory cytokines	IL-4	6.1 (± 8.3)	99.9 (± 0.0)	99.9 (± 0.0)	55.9 (± 16.0)
	IL-2	1.8 (± 5.0)	99.4 (± 0.2)	99.3 (± 0.3)	61.6 (± 13.6)
	IL-10	8.9 (± 7.7)	99.0 (± 0.4)	99.8 (± 0.0)	40.6 (± 14.9)
	IL-13	12.2 (± 5.9)	93.5 (± 0.0)	94.2 (± 0.0)	73.7 (± 20.6)
	IL-1Ra	11.2 (± 3.6)	90.2 (± 2.8)	92.1 (± 0.0)	35.4 (± 16.2)
	IL-12 p70	7.0 (± 7.5)	22.1 (± 4.5)	76.5 (± 2.5)	6.9 (± 8.0)
Complement factors	C3a	14.8 (± 11.5)	96.4 (± 1.2)	98.2 (± 0.2)	67.9 (± 7.5)
	C5a	1.3 (± 4.8)	90.7 (± 0.6)	95.7 (± 1.2)	38.8 (± 7.9)
Serine protease	PAI-1	8.5 (± 4.9)	87.9 (± 1.8)	95.5 (± 0.4)	30.9 (± 9.6)
Growth factors	FGF-23	18.3 (± 12.2)	98.7 (± 1.1)	99.4 (± 0.8)	88.4 (± 5.5)
	FGF-21	2.9 (± 0.5)	96.0 (± 0.9)	99.9 (± 0.0)	70.9 (± 10.1)
	G-CSF	8.1 (± 9.0)	36.0 (± 2.9)	99.4 (± 0.0)	16.9 (± 8.0)
Fluid removal	CRRT	–	Yes	No	No

*Abbreviations*: *C* complement, *CRRT* continuous renal replacement therapy, *FGF* fibroblast growth factor, *G-CSF* granulocyte colony-stimulating factor (glycoprotein), *HMGB-1* high-mobility group box 1 protein, *IL* interleukin, *IFN* interferon, *IP* interferon-induced protein, *LPS* lipopolysaccharide, *MCP* monocyte chemoattractant protein, *MIF* macrophage migration inhibitory factor, *MIP* macrophage inflammatory protein, *PAI* plasminogen activator inhibitor, *SD* standard deviation, *TNF* tumor necrosis factor, *Ra* receptor agonist, *α* alpha, *β* beta, *γ* gamma

#### Cytokines

For oXiris and CytoSorb, RRs of the pro- and anti-inflammatory cytokines studied were in the same ranges for 18 of the 20 mediators investigated (Table 2; Additional file 4: Figure S2). The RRs of > 70% for oXiris and > 80% for CytoSorb were observed for most of the pro- and anti-inflammatory mediators. The only exception was the lower RRs for oXiris vs CytoSorb for interleukin 12 (IL-12 p70; 22.1 ± 4.5% vs 76.5 ± 2.5%, respectively).

The RRs of all 14 pro-inflammatory mediators were statistically lower with Toraymyxin vs oXiris and CytoSorb, except for MIF (*p* = 0.103) and MIP-1A (*p* = 0.87; RR of the six inflammatory mediators were statistically lower with Toraymyxin vs oXiris and CytoSorb, except for IL-13 (*p* = 0.055) (Table 2; Additional file 4: Figure S2). Adsorption clearances and kinetics for each of the pro- and anti-inflammatory mediators investigated are shown in Fig. 4 and Additional file 3: Figure S1. In general, adsorption kinetics were faster with CytoSorb vs oXiris. However, for most mediators, t120 levels were comparable, and no significant differences were seen between CytoSorb and oXiris. The only exceptions were for IL-12 p70 and IL-1β, which were lower (indicating greater removal) with CytoSorb than with oXiris at t120 (69.0 ± 9.6 vs 238.4 ± 36.1 pg/ml and 0.8 ± 0 vs 3.8 ± 0.2 pg/ml, respectively).

#### Other inflammatory mediators

The RRs of complement factors (C3a and C5a), serine proteases (plasminogen activator inhibitor; PAI-1), fibroblast growth factors (FGF-23 and FGF-21), and glycoproteins (G-CSF) were higher with CytoSorb and oXiris vs Toraymyxin (Table 2; Additional file 4: Figure S2). RRs were in the same range for oXiris and CytoSorb for three of the six mediators but were slightly lower with oXiris for C5a (90.7 ± 0.6% vs 95.7 ± 1.2%, respectively) and PAI-1 (87.9 ± 1.8% vs 95.5 ± 0.4%) and significantly lower with oXiris for G-CSF (36.0 ± 2.9% vs 99.4 ± 0.0%).

RRs of all six factors (C3a, C5a, PAI-1, FGF-23, FGF-21, and G-CSF) were lower with Toraymyxin vs oXiris and CytoSorb. Differences were significant for all markers except for G-CSF which was only effectively adsorbed by CytoSorb (RR 99.4%; Table 2; Additional file 4: Figure S2). The kinetic removal profiles of the complement factors (C3a and C5a) and FGF-23 were comparable between oXiris and CytoSorb. FGF-21 and PAI-1 were initially removed more rapidly with CytoSorb, but the levels were comparable by 120 min (Fig. 4; Additional file 3: Figure S1).

### Mechanism of adsorption

**Fig. 5:**
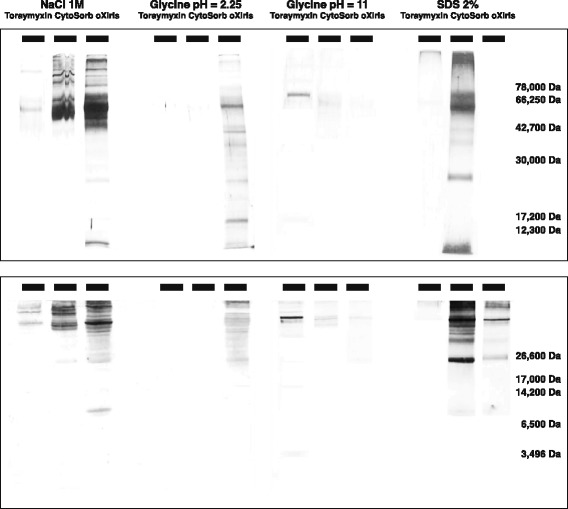
Electrophoretic patterns of membrane elutes. Upper panel is a Tris-glycine gel; lower panel is a Tris-tricine gel

Figure 5 shows the pattern of proteins eluted from the devices using different patterns (Tris-glycine on top and Tris-tricine on bottom) and buffers to differentiate ionic (NaCl 1 M and glycine pH 2.25 buffers) from hydrophobic (glycine pH 11 and SDS 2% buffer) mechanisms. Strong bands can be seen with the NaCl 1 M and pH 2.25 glycine buffers in the eluted fraction from oXiris and in the eluted fraction from the SDS buffer for CytoSorb. Strong bands were not detected with the eluted fractions from any of the four buffers for Toraymyxin.

## Discussion

In this study, we have shown that the three most widely available sorbent devices have very different spectrums of solute removal. While Toraymyxin is efficient in removing endotoxins, it does not effectively remove inflammatory mediators. Conversely, while Cytosorb removes a wide range of inflammatory mediators, it does not remove endotoxins. oXiris, by contrast, has similar adsorbent characteristics to Toraymyxin for endotoxins and to Cytosorb for most inflammatory mediators.

### Endotoxin removal

We observed RRs for endotoxins comparable with previous in vitro and clinical studies [13, 14]. Harm and colleagues found a reduction in LPS activity of 60 ± 14% with Toraymyxin using fresh human whole blood in vitro [14], while Romaschin and colleagues found a reduction in LPS activity of 88% with Toraymyxin using bovine serum or plasma in vitro [13]. In a systematic review of 28 studies of direct hemoperfusion with Toraymyxin, endotoxin levels decreased by 33–80% after Toraymyxin hemoperfusion in the 17 studies measuring such levels [15]. Overall, this review concluded that selective LPS-adsorption was associated with improvements in clinical outcomes (significant increases in mean arterial blood pressure, + 19 mmHg; reductions in vasoactive dopamine/dobutamine dose, − 1.8 μg/kg/min; and increases in gas exchange, mean arterial partial pressure of oxygen/fraction of inspired oxygen ratio, + 32 units) and in mortality risk (risk ratio, 0.53; 95% CI, 0.43–0.65). More recently, improvements in hemodynamic indices and survival rates have also been shown following Toraymyxin hemoperfusion of patients with severe sepsis or septic shock in the randomized controlled EUPHAS trial vs conventional therapy [15], the EUPHAS 2 registry [16], and in a prospective study [17]. However, no improvement was seen in either mortality or organ failure with Toraymyxin in a study enrolling 243 patients from 18 French intensive care units [18].

There is a paucity of data on the clinical effects of oXiris [19], and studies are ongoing [20]. However, the clinical benefits from the use of oXiris have been reported in a small study that found expedited improvement in organ function with oXiris vs conventional continuous venovenous hemofiltration only [21]. The total Sequential Organ Failure Assessment score improved significantly from baseline to 48 h in the oXiris group (*p* = 0.011; control *p* = 0.515), decreasing by 37% vs an increase of 3% in the control group (*p* = 0.013) [21].

We confirmed high endotoxin removal capacity with both oXiris and Toraymyxin over 6 h (oXiris 6.9 μg vs Toraymyxin 9.7 μg; total LPS quantity introduced was approximately 15.8 μg). High endotoxin plasma load in septic human blood has been previously reported in the range of 3–30 μg [13], supporting the clinically relevant removal capabilities of both devices.

### Pro- and anti-inflammatory mediators

Increased circulating levels of both pro-inflammatory (IL-6) and anti-inflammatory (IL-10) mediators have been associated with poor survival in numerous studies in patients with sepsis, including patients with septic shock randomized to different fluid resuscitation strategies [4]. Both IL-6 and IL-10 were effectively removed by oXiris (RR > 90%) and CytoSorb (RR > 90%), but RRs were lower for Toraymyxin (< 50%). RRs observed here for IL-10 (RR > 95% with CytoSorb) differ from the review by Honore and colleagues, who noted that CytoSorb was unable to capture IL-10 in humans [22], but are consistent with preclinical studies by Kellum and colleagues [23].

Elevated levels of C3a and C5a have been reported in sepsis, and high levels of C3a are associated with a higher risk of death [8, 24]. In our study, oXiris and CytoSorb displayed a significantly higher reduction of C3a and C5a (*p* < 0.05) compared with Toraymyxin. Additionally, greater removal of PAI-1 was found with oXiris and CytoSorb vs Toraymyxin. Levels of PAI-1, a marker of vascular endothelial cell activation and a major regulator of fibrinolysis, are increased by endotoxins, altering the normal balance between blood coagulation and fibrinolysis [5]. Following hemoperfusion, particularly with oXiris and CytoSorb, PAI-1 levels are decreased, modulating fibrinolysis and reducing the risk of sepsis-associated thrombosis formation. A recent study also indicated that the role of oXiris in fibrinolysis modulation may warrant further investigation [25]. Significant heterogeneity of inflammatory mediator levels in patients with sepsis/septic shock numerous investigators [4, 26]. Differential removal characteristics might therefore be advantageous and lead to a more personalized approach to medicine.

We observed that the results are consistent with the nature of the devices investigated; oXiris is a highly electrically charged hydrogel, while CytoSorb is a hydrophobic resin. However, previous studies using molecular mechanic simulations have shown that the binding of polymyxin B to endotoxins occurs through hydrophobic bonds between fatty acid chains of the lipid A in endotoxin and hydrophobic amino acids of polymyxin B and through ionic interactions between the endotoxin’s negatively charged phosphate groups in lipid A and the positively charged amino groups of polymyxin B [5, 27]. Toraymyxin is a device made of rolled woven fibers, whereby the blood circulates at the external side of the fibers [10]. From a design perspective, the accessible surface area for adsorption is most probably much lower as compared with CytoSorb, which is made of small polymer beads [11]. The oXiris device also benefits from a large surface area available for adsorption [9], the bulk membrane being completely dense and isotropic. In the case of Toraymyxin, the proportion of charged groups at the surface to accessible remaining hydrophobic residues is also unknown, and both chemical groups may compete.

Our study has limitations that should be acknowledged. First, as an in vitro study, the outcomes observed here may not be representative of the clinical setting. Second, the experimental setup used human plasma that had been preincubated with the mediators. This system does not allow us to examine the impact of each device on the inflammatory response itself, i.e., the generation and release of the inflammatory mediators during and after the treatment, as well as potential interactions with blood cells. Furthermore, we cannot exclude the possibility of competition between the different mediators for adsorption, given they were added simultaneously in each experiment. However, the high reproducibility observed between experiments, combined with the relatively low quantities of soluble toxins introduced, suggests that limited competition occurred in the experimental setup. Finally, the concentrations used were pathological, and our data can only be used to comment on the adsorption of these mediators at these concentrations.

## Conclusions

oXiris was shown to have the broadest adsorption capacity of the devices tested. oXiris displayed similar adsorption to Toraymyxin for endotoxin removal and similar adsorption to CytoSorb for the removal of most cytokines and other inflammatory mediators. Further investigation is warranted as to whether a more continuous blood purification with oXiris may improve outcomes in terms of reducing immune cell activation and organ damage compared to short-term intended use (e.g., Toraymyxin use is limited to 2 h).

## Additional files

Additional file 1: **Table S1.** Pathological concentrations of mediators included in the study. (DOCX 25 kb)

Additional file 2: **Table S2.** Inflammatory mediators included in the study. (DOCX 30 kb)

Additional file 3: **Figure S1.** Kinetic removal profiles of a) endotoxin, b) pro-inflammatory cytokines, c) anti-inflammatory cytokines, and d) other inflammatory mediators. (DOCX 867 kb)

Additional file 4: **Figure S2.** Removal ratios of a) pro-inflammatory cytokines, b) anti-inflammatory cytokines, and c) other inflammatory mediators. (DOCX 148 kb)

## Abbreviations

AKI: Acute kidney injury
C: Complement
CRRT: Continuous renal replacement therapy
FGF: Fibroblast growth factor
G-CSF: Granulocyte colony-stimulating factor
HMGB: High-mobility group box 1 protein
IFN: Interferon
IL: Interleukin
IP: Interferon-induced protein
LPS: Lipopolysaccharide
MCP: Monocyte chemoattractant protein
MIF: Macrophage migration inhibitory factor
MIP: Macrophage inflammatory protein
PAI: Plasminogen activator inhibitor
RR: Removal rates
TNF: Tumor necrosis factor
α: Alpha
β: Beta
γ: Gamma
