# Supplementary material for: In vitro comparison of the adsorption of inflammatory mediators by blood purification devices
Source: Intensive Care Med Exp. 2018 May 4;6:12. doi: 10.1186/s40635-018-0177-2 (PMC5935601; doi:10.1186/s40635-018-0177-2)
Supplement: Supplementary file 1 — Table S1. Pathological concentrations of mediators included in the study. (DOCX 25 kb) [file 40635_2018_177_MOESM1_ESM.docx]

**Additional file 1**

**Table S1** Pathological concentrations of mediators included in the study

|  |  | *In vitro experiment* | |
| --- | --- | --- | --- |
| Mediators | Mediators plasma concentration reported for | Experimental pool concentration  *Pool volume of each experiment = 500ml* | Corresponding theoretical pool concentration  *Extrapolated to a human plasma volume = 2.5L* |
| LPS (EU/ml) | > 10 [1] | [25 – 75]* | [5 – 15] |
| IL1-ra (pg/ml) | 1,200 (236.8–50,700) [2] | 1500 | 300 |
| IL1-β (pg/ml) | 23.5 (0.0–244.3) [2]  1.22 (0.01–7.33) [3] | 100 | 20 |
| IL-2 (pg/ml) | 1. (0.0–102.5) [2]   300 ± 700 [4] | 500 | 100 |
| IL-3 (pg/ml) | 89.4 (0.0 – 550) [5] | 1500 | 300 |
| IL-4 (pg/ml) | 142.8 (122.2–156.8) [2] | 500 | 100 |
| IL-6 (pg/ml) | 126.3 (0.0–910.7) [2]  157 (68–575) [6]  351 (211–584) [7]  420 (380–430) [8] | 1500 | 300 |
| IL-8 (pg/ml) | 197.9 (0.0–4,850) [2]  145.3 (74.37–520.2) [3] | 1500 | 300 |
| IL-10 (pg/ml) | 0.0 (0.0–238.9) [2]  27.45 (6.83–116.3) [3]  3.4 (0.5–28.2) [6]  53 (7–169) [9]  23 (13–40) [7]  58 (19–140) [8] | 500 | 100 |
| IL-12 (pg/ml) | 774.6 (660.9–1,431) [2]  33.29 (0.0 – 310.85) [10] | 500 | 100 |
| IL-13 (pg/ml) | 7.21 (0.03–19.29) [3] | 100 | 20 |
| IL-17A (pg/ml) | 118.8 (102.0–142.5) [2] | 500 | 100 |
| MCP-1 (pg/ml) | 523.9 (117.9–1,723) [2]  753.9 (324.6–1689) [3]  710 (520–910) [8] | 5000 | 1000 |
| MIP-1α (pg/ml) | 56.8 (51.7–1,749) [2] | 500 | 100 |
| MIP-1β (pg/ml) | 67.5 (51.3–3,531) [2] | 500 | 100 |
| G-CSF (pg/ml) | 0.0 (0.0–57.0) [2]  640 (450–950) [8] | 1500 | 300 |
| INFγ (pg/ml) | 67.2 (60.0–78.4) [2]  33.10 (0.00–116.7) [3] | 250 | 50 |
| IP-10 (pg/ml) | 510 (180–1000) [8] | 5000 | 1000 |
| TNF-α (pg/ml) | 14.46 (2.68–47.00) [3]  68 (36–143) [6]  39 (18–79) [9] | 250 | 50 |
| Eotaxin (pg/ml) | 68.6 (35.4–172.9) [2] | 500 | 100 |
| HMGB-1 (ng/ml) | 2.2 (0.9–3.8) [11]  8.3 ± 10.1 [12]  34 ± 76 [13] | 30 | 6 |
| FGF-21 (pg/ml) | 3900 (400–4400) [14] | 7500 | 1500 |
| FGF-23 (pg/ml) | 2942 (448–5068) [15] | 7500 | 1500 |
| C3a (pg/ml) | 1450.89 ± 352.11 [16] | 400 | 80** |
| C5a (ng/ml) | 5.24 ± 3.80 [16] | 15 | 3 |
| MIF (pg/ml) | 5616.4 (1547.9–10275.4) [17]  2470 (1560–6339) [18] | 350 | 70** |
| PAI-1 (ng/ml) | 33.1 (18.6–63.6) [19]  31.7 (21.2–41.6) [20] | 1.5 | 0.3** |

***Abbreviations: C*3*a* complement 3a, *C5a* complement 5a, *FGF* fibroblast growth factor, *G-CSF* granulocyte-colony stimulating factor, *HMGB-1* high-mobility group box 1 protein, *IL* interleukin, *IFN* interferon, *IP* interferon-induced protein, *MCP* monocyte chemoattractant protein, *MIF* macrophage migration inhibitory factor, *MIP* macrophage inflammatory protein, *PAI* plasminogen activator inhibitor, *TNF* tumor necrosis factor, *Ra* receptor agonist, *α* alpha; *β* beta, *γ* gamma**

*Targeted range after pool stabilization.

**For feasibility reasons, those mediators could not be challenged in pathological quantities.

**References**

1. Foster D, Derzko A, Romaschin A (2004) A novel method for rapid detection of human endotoxaemia, as published in CLI.

2. Lvovschi V, Amaud L, Parizot C, Freund Y, Juillien G, Ghillani-Dalbin P, Bouberima M, Larsen M, Riou B, Gorochov G, Hausfater P (2011) Cytokine Profiles in Sepsis Have Limited Relevance for Stratifying Patients in the Emergency Department: A Prospective Observational Study. PLoS ONE 6: e28870.

3. Bozza FA, Salluh JI, Japiassu AM, Soares M, Assis EF, Gomes RN, Bozza MT, Castro-Faria-Neto HC, Bozza PT (2007) Cytokine profiles as markers of disease severity in sepsis: a multiplex analysis Critical Care 11:R49.

4. Pinsky MR, Vincent JL, Deviere J, Alegre M, Kahn RJ, Dupont E (1993) Serum cytokine levels in human septic shock. Relation to multiple-system organ failure and mortality Chest 103:565‒575.

5. Weber F, Chousterman BG, He S, Fenn AM, Nairz M, Anzai A, Brenner T, Uhle F, Iwamoto Y, Robbins CS, Noiret L, Maier SL, Zönnchen T, Rahbari NN, Schölch S, Klotzsche-von Ameln A, Chavakis T, Weitz J, Hofer S, Weigand MA, Nahrendorf M, Weissleder R, Swirski FK. (2015) Interleukin-3 amplifies acute inflammation and is a potential therapeutic target in sepsis Science 347:1260‒1265.

6. Simmons EM, Himmelfarb J, Sezer MT, Chertow GM, Mehta RL, Paganini EP, Soroko S, Freedman S, Becker K, Spratt D, Shyr Y, Ikizler TA; PICARD Study Group (2004) Plasma cytokine levels predict mortality in patients with acute renal failure Kidney International 65:1357–1365.

7. Hoogerwerf JJ, Tanck MW, van Zoelen MA, Wittebole X, Laterre PF, van der Poll T (2010) Soluble ST2 plasma concentrations predict mortality in severe sepsis Intensive Care Med 36:630-637.

8. Fjell CD, Thair S, Hsu JL, Walley KR, Russell JA, Boyd J (2013) Cytokines and Signaling Molecules Predict Clinical Outcomes in Sepsis PLoS ONE 8:e79207.

9. Lorente L, Martín MM, Varo N, Borreguero-León JM, Solé-Violán J, Blanquer J, Labarta L, Díaz C, Jiménez A, Pastor E, Belmonte F, Orbe J, Rodríguez JA, Gómez-Melini E, Ferrer-Agüero JM, Ferreres J, Llimiñana MC, Páramo JA (2011) Association between serum soluble CD40 ligand levels and mortality in patients with severe sepsis Critical Care 15:R97.

10. Wu HP, Chen CK, Chung K, Tseng JC, Hua CC, Liu YC, Chuang DY, Yang CH (2009) Serial cytokines levels in patients with severe sepsis Inflammation research 58:358‒393.

11. Gaïni S, Pedersen SS, Koldkjaer OG, Pedersen C, Møller HJ (2007) High mobility group box-1 protein in patients with suspected community-acquired infections and sepsis: a prospective study Critical Care 11:R32.

12. Hatada T, Wada H, Nobori T, Okabayashi K, Maruyama K, Abe Y, Uemoto S, Yamada S, Maruyama I (2005) Plasma concentrations and importance of High Mobility Group Box protein in the prognosis of organ failure in patients with disseminated intravascular coagulation Thrombosis and haemostasis 94:975‒979.

13. Sundén-Cullberg J, Norrby-Teglund A, Rouhiainen A, Rauvala H, Herman G, Tracey KJ, Lee ML, Andersson J, Tokics L, Treutiger CJ (2005) Persistent elevation of high mobility group box-1 protein (HMGB1) in patients with severe sepsis and septic shock Crit Care Med 33:564–573.

14. Gariani K, Drifte G, Dunn-Siegrist I, Pugin J, Jornayvaz FR (2013) Increased FGF21 plasma levels in humans with sepsis and SIRS, Endocrine Connections 2:146–153.

15. Leaf DE, Wolf M, Waikar SS, Chase H, Christov M, Cremers S, Stern L (2012) FGF-23 levels in patients with AKI and risk of adverse outcomes, Clin J Am Soc Nephrol 7:1217‒1223.

16. Helling H, Stephan B, Pindur G (2015) Coagulation and complement system in critically ill patients Clinical Hemorheology and Microcirculation 61:185–193.

17. Chuang TY, Chang HT, Chung KP, Cheng HS, Liu CY, Liu YC, Huang HH, Chou TC, Chang BL, Lee MR, Lin CJ, Lee SW, Yu CJ, Hsueh PR (2014) High levels of serum macrophage migration inhibitory factor and interleukin 10 are associated with a rapidly fatal outcome in patients with severe sepsis International Journal of Infectious Diseases 20:13–17.

18. Bozza A, Gomes RN, Japiassú AM, Soares M, Castro-Faria-Neto HC, Bozza PT, Bozza MT (2004) Macrophage migration inhibitory factor levels correlate with fatal outcome in sepsis Shock 22:309–313.

19. Lorente L, Martín MM, Borreguero-León JM, Barrios Y, Solé-Violán J, Ferreres J, Labarta L, Díaz C, Jiménez A. (2015) The 4G/4G Genotype of PAI-1 Polymorphism Is Associated with Higher Plasma PAI-1 Concentrations and Mortality in Patients with Severe Sepsis PLoS ONE 10:e0129565.

20. Panigada M, Zacchetti L, L'Acqua C, Cressoni M, Anzoletti MB, Bader R, Protti A, Consonni D, D'Angelo A, Gattinoni L (2015) Assessment of Fibrinolysis in Sepsis Patients with Urokinase Modified Thromboelastography PLoS ONE 10:e0136463.
