# Supplementary material for: In vitro comparison of the adsorption of inflammatory mediators by blood purification devices
Source: Intensive Care Med Exp. 2018 May 4;6:12. doi: 10.1186/s40635-018-0177-2 (PMC5935601; doi:10.1186/s40635-018-0177-2)
Supplement: Supplementary file 2 — Table S2. Inflammatory mediators included in the study. (DOCX 30 kb) [file 40635_2018_177_MOESM2_ESM.docx]

**Additional file 2**

**Table S2** Inflammatory mediators included in the study

| Substance class | Substance name | Molecular weight (Da) | Isoelectric point |
| --- | --- | --- | --- |
| Endotoxins | LPS |  |  |
| Chemokines | MIP-1α | 10,000 | 4.77 |
|  | MIP-1β | 10,200 | 5.13 |
|  | Eotaxin | 10,700 | 9.98 |
|  | MCP-1 | 11,000 | 9.40 |
|  | IP-10 | 10,900 | 9.93 |
|  | IL-8 | 8,900 | 9.24 |
| Cytokines | IL-1β | 30,700 | 4.70 |
| Pro-inflammatory | IL-6 | 23,800 | 6.17 |
|  | IL-12 p70 | 70,000 | 6.20–7.00 |
|  | IL-17A | 17,500 | 8.82 |
|  | MIF | 12,500 | 7.73 |
|  | TNF-α | 54,000 | 7.00 |
| Cytokines | IL-1Ra | 19,900 | 4.73 |
| Anti-inflammatory | IL-4 | 17,500 | 9.17 |
|  | IL-10 | 20,500 | 8.19 |
|  | IL-13 | 15,800 | 8.34 |
| Cytokines | IL-2 | 17,600 | 7.67 |
| Immunoregulatory | IFNɣ | 19,300 | 9.5 |
|  | IL-3 | 17,200 | 8.69 |
| Growth factors | G-CSF | 22,300 | 5.61 |
|  | FGF-21 | 22,300 | 5.01 |
|  | FGF-23 | 27,900 | 9.17 |
| Complement factors | C5a | 8,300 | 8.93 |
|  | C3a | 9,100 | 9.69 |
| Homeostasis markers | PAI-1 | 45,000 | 6.68 |
| High mobility group | HMGB-1 | 24,900 | 5.62 |

***Abbreviations: C*3*a* complement 3a, *C5a* complement 5a, *FGF* fibroblast growth factor, *G-CSF* granulocyte-colony stimulating factor, *HMGB-1* high-mobility group box protein, *IL* interleukin, *IFN* interferon, *IP* interferon-induced protein, *LPS* lipopolysaccharide, *MCP* monocyte chemoattractant protein, *MIF* macrophage migration inhibitory factor, *MIP* macrophage inflammatory protein, *PAI* plasminogen activator inhibitor, *TNF* tumor necrosis factor, *Ra* receptor agonist, *α* alpha, *β* beta, *γ* gamma**
