# Supplementary material for: In vitro comparison of the adsorption of inflammatory mediators by blood purification devices
Source: Intensive Care Med Exp. 2018 May 4;6:12. doi: 10.1186/s40635-018-0177-2 (PMC5935601; doi:10.1186/s40635-018-0177-2)
Supplement: Supplementary file 3 — Figure S1. Kinetic removal profiles of a) endotoxin, b) pro-inflammatory cytokines, c) anti-inflammatory cytokines, and d) other inflammatory mediators. (DOCX 867 kb) [file 40635_2018_177_MOESM3_ESM.docx]

**Additional file 3**

**Figue S1.** Kinetic removal profiles of a) endotoxin, b) pro-inflammatory cytokines, c) anti-inflammatory cytokines, and d) other inflammatory mediators

**a) Endotoxin**

| **LPS**  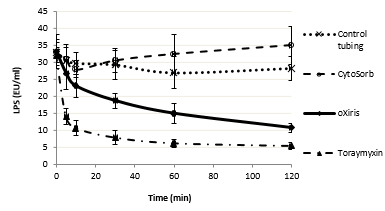 |  |  |
| --- | --- | --- |

**b) Pro-inflammatory cytokines**

| **IL-6**  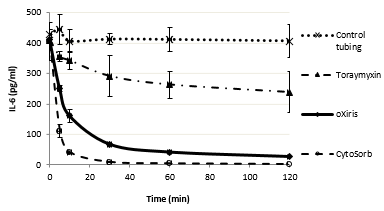 | **Eotaxin**  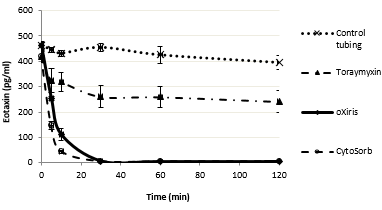 | **MCP-1**  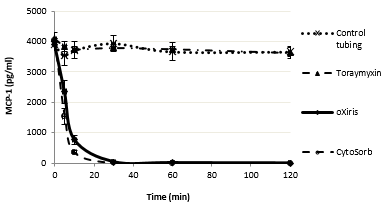 |
| --- | --- | --- |
| **TNF-α**  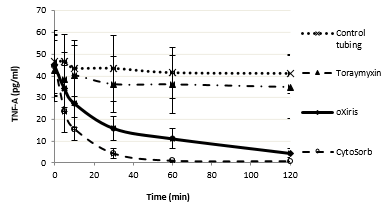 | **IL-17α**  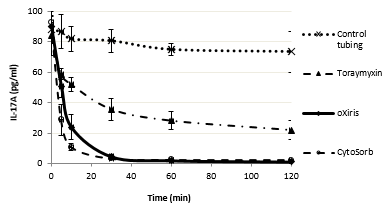 | **IL-1β**  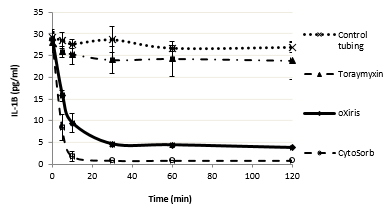 |

| **HMGB-1**  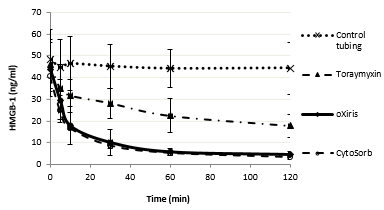 | **IFN-γ**  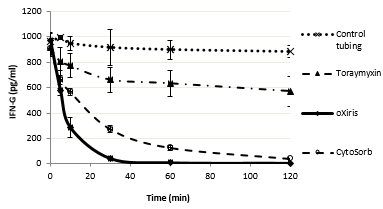 | **IL-8**  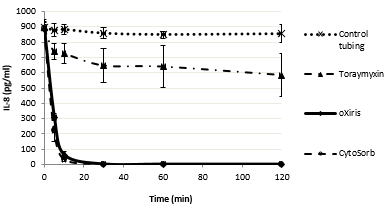 |
| --- | --- | --- |
| **MIF**  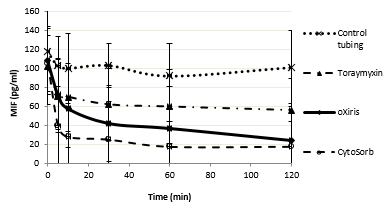 | **MIP-1α**  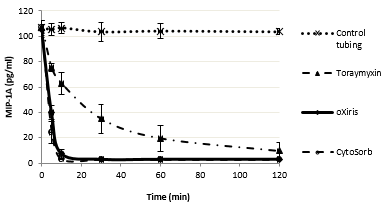 | **IL-3**  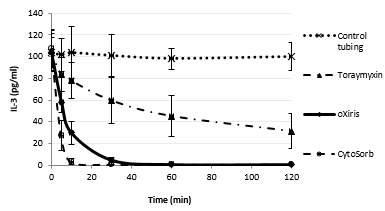 |
| **IP-10**  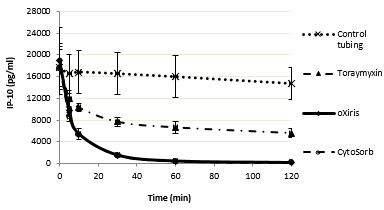 | **MIP-1β**  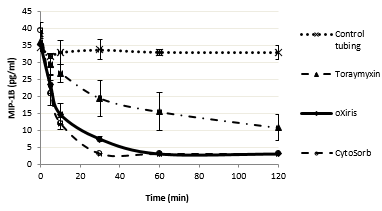 |  |

**c) Anti-inflammatory cytokines**

| **IL-4**  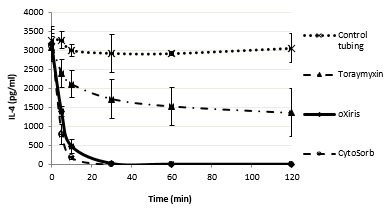 | **IL-13**  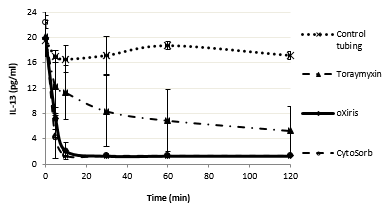 | **IL-2**  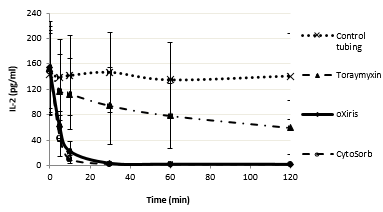 |
| --- | --- | --- |
| **IL-10**  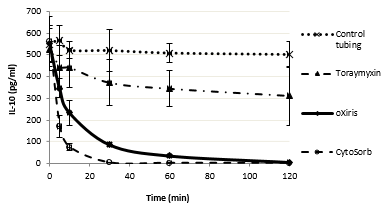 | **IL-1Ra**  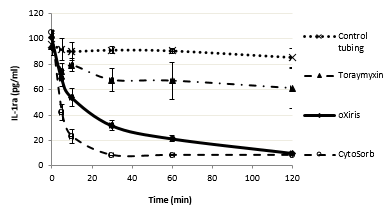 | **IL-12 p70**  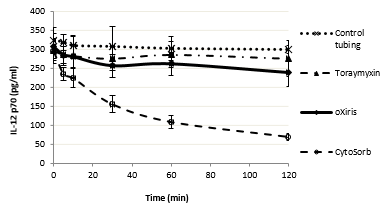 |

**d) Other inflammatory mediators**

| **C5a**  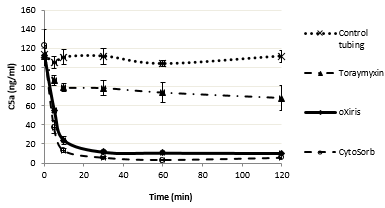 | **C3a**  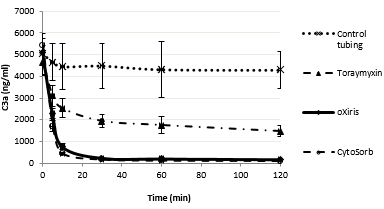 | **PAI-1**  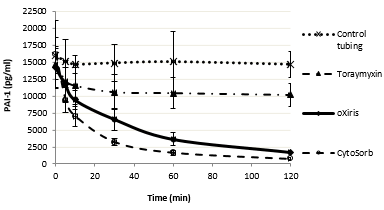 |
| --- | --- | --- |
| **FGF-23**  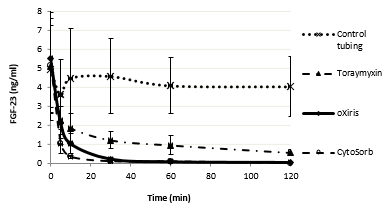 | **FGF-21**  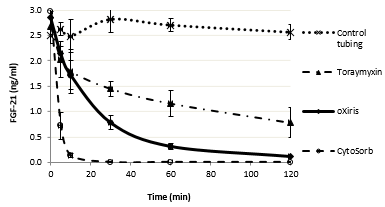 | **G-CSF**  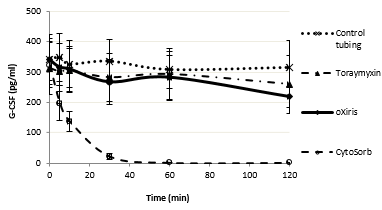 |

***Abbreviations: C*3*a* complement 3a, *C5a* complement 5a, *FGF* fibroblast growth factor,** ***G-CSF* granulocyte-colony stimulating factor, HMGB-1 high-mobility group box 1 protein, *IL* interleukin, *IFN* interferon,** ***IP* interferon-induced protein,** ***LPS* lipopolysaccharide, *MCP* monocyte chemoattractant protein, *MIF* macrophage migration inhibitory factor, *MIP* macrophage inflammatory protein, *PAI* plasminogen activator inhibitor, *TNF* tumor necrosis factor, *Ra* receptor agonist,** α **alpha, *β* beta, *γ*** **gamma**
