# Supplementary material for: In vitro comparison of the adsorption of inflammatory mediators by blood purification devices
Source: Intensive Care Med Exp. 2018 May 4;6:12. doi: 10.1186/s40635-018-0177-2 (PMC5935601; doi:10.1186/s40635-018-0177-2)
Supplement: Supplementary file 4 — Figure S2. Removal ratios of a) pro-inflammatory cytokines, b) anti-inflammatory cytokines, and c) other inflammatory mediators. (DOCX 148 kb) [file 40635_2018_177_MOESM4_ESM.docx]

**Additional file 4**

**Figure S2.** Removal ratios of a) pro-inflammatory cytokines, b) anti-inflammatory cytokines, and c) other inflammatory mediators

**a) Pro-inflammatory cytokines**

| **IL-6** | **Eotaxin** | **MCP-1** |
| --- | --- | --- |

| **TNF-α** | **IL-17α** | **IL-1β** |
| --- | --- | --- |
| **HMGB-1** | **IFN-γ** | **IL-8** |

| **MIF** | **MIP-1α** | **IL-3** |
| --- | --- | --- |
| **IP-10** | **MIP-1β** |  |

**b) Anti-inflammatory cytokines**

| **IL-4** | **IL-13** | **IL-2** |
| --- | --- | --- |
| **IL-10** | **IL-1Ra** | **IL-12 p70** |

**c) Other inflammatory mediators**

| **C5a** | **C3a** | **PAI-1** |
| --- | --- | --- |
| **FGF-23** | **FGF-21** | **G-CSF** |

***Abbreviations: C*3*a* complement 3a, *C5a* complement 5a, *FGF* fibroblast growth factor, *G-CSF* granulocyte-colony stimulating factor, *HMGB-1* high-mobility group box 1 protein, *IL* interleukin, *IFN* interferon, *IP* interferon-induced protein, *MCP* monocyte chemoattractant protein, *MIF* macrophage migration inhibitory factor, *MIP* macrophage inflammatory protein, *PAI* plasminogen activator inhibitor, *TNF* tumor necrosis factor, *Ra* receptor agonist, *α* alpha; *β* beta, *γ* gamma**
